# Iterative Nonlocal Total Variation Regularization Method for Image Restoration

**DOI:** 10.1371/journal.pone.0065865

**Published:** 2013-06-11

**Authors:** Huanyu Xu, Quansen Sun, Nan Luo, Guo Cao, Deshen Xia

**Affiliations:** School of Computer Science and Technology, Nanjing University of Science and Technology, Nanjing, Jiangsu, China; Institute of Psychology, Chinese Academy of Sciences, China

## Abstract

In this paper, a Bregman iteration based total variation image restoration algorithm is proposed. Based on the Bregman iteration, the algorithm splits the original total variation problem into sub-problems that are easy to solve. Moreover, non-local regularization is introduced into the proposed algorithm, and a method to choose the non-local filter parameter locally and adaptively is proposed. Experiment results show that the proposed algorithms outperform some other regularization methods.

## Introduction

Image restoration is a classical inverse problem which has been extensively studied in various areas such as medical imaging, remote sensing and video or image coding. In this paper, we focus on the common image acquisition model: an ideal image 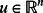 is observed in the presence of a spatial invariance blur kennel 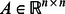 and a additive Gaussian white noise 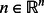 with zero mean and standard deviation 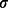. Then, the observed image 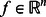 is obtained by:
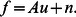
(1)


Restoring the ideal image from the observed image is ill-posed since the blur kennel matrix is always singular. A common way to solve this problem is to use regularization methods, in which regularization terms can be invited to restrict the solutions. Regularization methods generally have the form as follows:
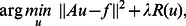
(2)where 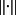 denotes the Euclidean norm, 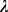 is a positive regularization parameter balancing the fitting term and the regularization term, 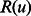 is the regularization term. The total variation regularization proposed by Rudin, Osher and Fatemi [1] (also called the ROF model) is a well known regularization method in this field. The total variation norm has a piecewise smooth regularization property, thus the total variation regularization can preserve edges and discontinuities in the image. The unconstrained ROF model has the form
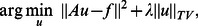
(3)where the term 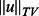 stands for the total variation of the image. The continuous form of the total variation is defined as



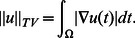
(4)Many numerical methods was proposed to solve (3). When 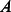 is an identity matrix, the ROF model (3) turns into a TV denoising problem, methods as Chambolle's projection method [2], semismooth Newton methods [3], multilevel optimization method [4] and split Bregman method [5]. When 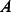 is a blur kennel matrix, (3) turns into a TV deblurring problem, we have prime-dual optimization algorithms for TV regularization [6]–[9], forward backward operator splitting method [10], interior point method [11], majorization-minimization approach for image deblurring [12], Bayesian framework for TV regularization and parameter estimation [13]–[15], using local information and Uzawa's algorithm [16], [17], using regularized locally adaptive kernel regression [18], augmented Lagrangian methods [19], [20] and so on. However, the problem is far from perfectly solved, problems as edge and detail preserving [21], [22], ringing effect reducing [23]–[25] and varied blur kennels and noise types image restoration [26], [27] still need better solutions.

The purpose of this paper is to propose an effective total variation minimization algorithm for image restoration. The algorithm is based on Bregman iteration which can give significant improvement over standard models [28]. Then, we solve the proposed algorithm by alternately solving a deblurring problem and a denoising problem [29], [30]. In addition, we propose a local adaptive nonlocal regularization approach to improve the restoration results.

The structure of the paper is as follows. In the next section, an iterative algorithm for total variation based image restoration is proposed, moreover we present a nonlocal regularization under the proposed algorithm framework with local adaptive filter parameter to improve the restoration results. Section Experiments shows the experimental results. Section Conculsions concludes this paper.

## Methods

### Iterative Approach for TV-based Image Restoration

#### Bregman iteration for image restoration

We first consider a general minimization problem as follows:
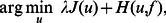
(5)where 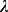 is the regularization parameter, 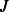 is a convex nonnegative regularization functional and the fitting functional 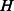 is convex nonnegative with respect to 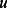 for fixed 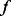. This problem is difficult to solve numerically when 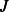 is non-differentiable, and the Bregman iteration is an efficient method to solve the minimization problem.

Bregman iteration is based on the concept of “Bregman distance”. The Bregman distance of a convex functional 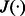 between points 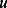 and 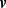 is defined as:
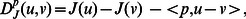
(6)where 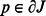 is a sub-gradient of 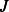 at the point 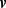. Bregman distance generally is not symmetric, so it is not a distance in the usual sense, but the Bregman distance measures the closeness of two points. 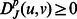 for any 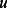 and 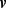, and 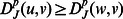 for all points 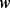 on the line segment connecting 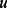 and 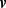. Using Bregman distance (6), the original minimization problem (5) can be solved by an iterative procedure:
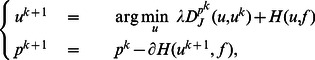
(7)where 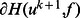 denotes a sub-gradient of 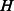 at 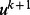 and 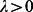. When we choose 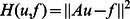 and 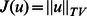, (7) turns into the total variation minimization problem, then (7) can be converted into the following two step Bregman iterative scheme [28]:



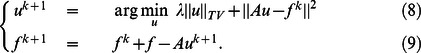



In [28], the authors mentioned that the sequence 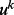 weakly converges to a solution of the unconstrained form of (5), and the sequence 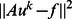 converges to zero monotonically. We can see from (8),(9) that,the Bregman iteration just turns the original problem (5) into a iteration procedure and add the noise residual back into the degenerated image at the end of every iteration. Bregman iteration converges fast and gets better results than standard methods. Bregman iteration was widely used in varied areas of image processing [5], [31]–[33].

#### General framework of the iterative algorithm

As introduced above, we use the Bregman iteration (8),(9) to bulid the main iterative framework. Rather than considering (3), we consider the problem as follows:
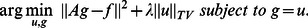
(10)


We separated the variable 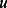 in (3) into two independent variables, so we can split the original problem (3) into sub-problems which are easy to solve. This problem is obviously equivalent to (3). We can replace (10) into the unconstrained form:
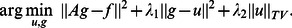
(11)



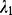
 and 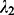 are regularization parameters balancing the three terms. If the regularization parameter 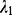 is big enough, the problem (11) is close to the problem (10), and the solutions of the problems are similar. If we let 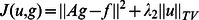 and 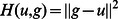, we can see that 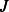 and 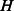 are all convex, and then (10) is a simple application of (7). Thus, the above problem can be solved by using Bregman iteration:
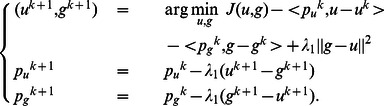
(12)


Similar to [28], we can reform the above procedure into a simple two step iteration algorithm:
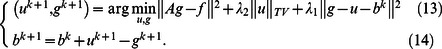



As we can see in (13), when 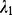 tends to infinity, the above algorithm is equal to the original Bregman iterative algorithm in [28]. we use an alternating minimization algorithm [29], [30] to solve (13). We split (13) into a deblurring and a denoising sub-problems. Thus, (13) can be solved by the following two step iterative formation:
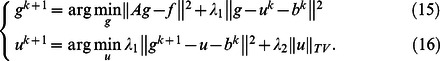



We can see that (15) is an 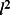-norm differentiable problem, we can solve it as follows:
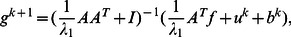
(17)where 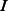 is the identity matrix and the matrix 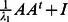 is invertible. Then (15) can be solved by optimization techniques such as Gauss-Seidel, conjugate gradient or Fourier transform. As for (16), it is a exact total variation denoising problem, we can use Chambolle's projection algorithm [2], semismooth Newton method [3] or split Bregman algorithm [5] to solve this problem.

Thus, the proposed alternating Bregman iterative method for image restoration can be formed as follows:

Algorithm 1: Alternating Bregman iterative method for image restoration.
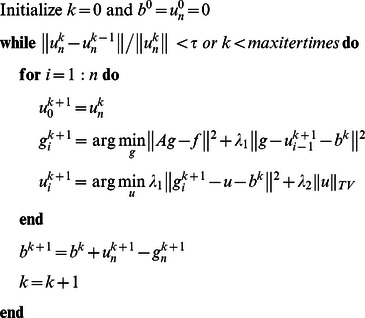



#### Analysis of the proposed algorithm

First, we show some important monotonicity properties of the Bregman iteration proposed in [28].

**Theorem 1** The sequence 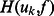 obtained from the Bregman iteration is monotonically nonincreasing. And assume that there exists a minimizer 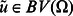 of 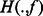 such that 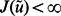. Then.
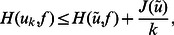
(18)and, in particular, 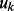 is a minimizing sequence.

Moreover, 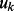 has a weak-* convergent subsequence in 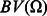, and the limit of each weak-* convergent subsequence is a solution of 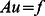. If 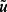 is the unique solution of 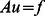, then 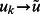 in the weak-* topology in 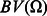.

Then, we show that the alternating minimization algorithm (15) and (16) also convergence to the solution of the sub-problem (13) [30]. Let 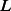 be the difference matrix and 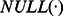 denotes the null space of the corresponding matrix, we obtain the following theorem.

**Theorem 2** For any initial guess 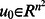, suppose 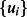 is generated by (15) and (16), then 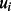 converges to a stationary point of (10). And when 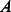 is a matrix of full column rank, 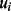 converges to a minimizer of (10).

Then, we can get the following convergence theorem of the proposed alternating Bregman iterative method.

**Theorem 3** Let 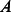 be a linear operator, consider the algorithm 1. Suppose 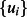 is a sequence generated by algorithm 1, 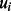 converges to a solution of the original constrained problem (3).

*Proof.* Let 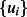 and 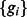 be the sequence obtained from (13), and every 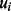 is the solution of the (13), moreover 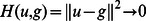 with the increase in iterations of the algorithm 1. Suppose in one iteration, there is 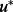 and 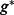 satisfying 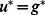, and let the true solutions of the problem (10) be 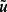 and 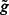, then.
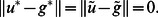
(19)


Due to 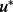 and 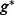 satisfy (11), 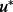 and 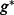 can enable the convex function (11) to obtain its Minimum value. Then.
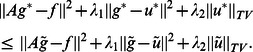
(20)


Thus, we can obtain.
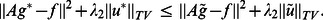
(21)


Owning to 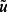 and 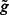 are the true solutions of the problem (10), this inequality implies that 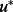 and 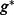 are also the solutions of the problem (10), thus are the solutions of the original unconstrained problem (3).

#### Connection with other methods

We noticed that the equation (17) can be rewrite as follows:
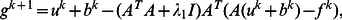
(22)thus, the proposed algorithm 1 can be interpreted as follows:



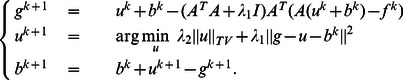
(23)The preconditioned Bregmanized nonlocal regularization (PBOS) algorithm [33] can be formed as:
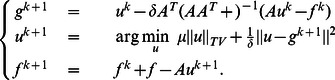
(24)the left and right pseudo inverse approximation are equal:



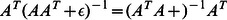
(25)Compare these two methods, we can see the only difference between them is the way to calculate the noise and add it back to the iteration. The PBOS method calculates the undeconvolutioned noise and only add it back to the calculation of g, while the proposed method calculate the deconvolutioned noise and add it back to both the calculations of g and u, we believe that is why the proposed algorithm have a faster converge speed and better restoration results according to the experiments in section 0.

### Adaptive Nonlocal Regularization

#### Nonlocal regularization

Recently, nonlocal methods have been extensively studied, the nonlocal means filter was first proposed by Buades et al [34]. The main idea of the nonlocal means denoising model is to denoise every pixel by averaging the other pixels with similar structures (patches) to the current one. Based on the nonlocal means filter, Kindermann et al. [35] tried to investigate the use of regularization functionals with nonlocal correlation terms for general inverse problems. Inspired by the graph Laplacian and the nonlocal means filter, Gilboa and Osher defined a variational framework based nonlocal operators [36]. In the following, we use the definitions of the nonlocal regularization functionals introduced in [36].

Let 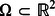, 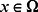, 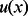 is a real function 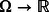 and 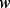 is a nonnegative symmetric weight function. Then the nonlocal gradient 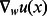 is defined as the vector of all partial differences 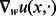 at 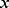:
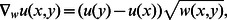
and the graph divergence 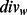 of a vector 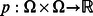 can be defined as:



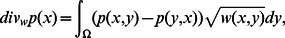
the weight function is defined as the nonlocal means weight function:
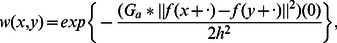
(26)where 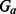 is the Gaussian kernel with standard deviation 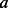, 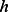 is the filtering parameter related to the standard variance of the noise, and the  in 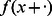 stands for a square patch centered by point 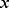. When the reference image 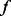 is known, the nonlocal means filter is a linear operator. The definition of the weight function (26) shows that the value of the weight is significant only when the patch around 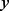 has similar structure as the corresponding patch around 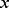.

The nonlocal TV norm can be defined as isotropic 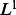 norm of the weighted graph gradient 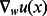:
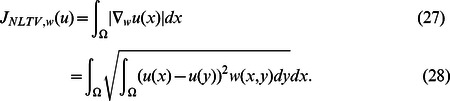



The main idea of the nonlocal regularization is to generalize the local gradient and divergence concepts to the nonlocal form. Then the nonlocal means filter is generalized to the variational framework.

The nonlocal means filter and the nonlocal regularization functionals can reduce noise efficiently and preserve textures and contrast of the image. Generally, it is good to choose a reference image as close as possible to the original ideal image to calculate the weights. However, the original image structures are broken in the degraded image, we can not get the precise weights between the pixels, thus the weights should be calculated from a preprocessed image [37]. In our alternating minimization framework, we get the deblurred image at the first step, then denoise the deblurred image at the second step. As the nonlocal regularization functionals are robust to the noise, and the structures of the deblurred image are close to the original ideal image, we can calculate the weights by using the deblurred image as the reference image, then apply a nonlocal denoising step to obtain the restored image.

#### Adaptive nonlocal parameter selection

Within the alternating Bregman iterative method, we can use 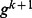 as the reference image to calculate the weights of the nonlocal regularization functionals, then use the weights to denoise the deblurred image 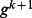 at every iteration. Note that the nonlocal filter parameter 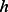 is related to the standard variance of the noise, however we do not know the exact noise of the image 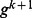. Moreover, when we use the single filter parameter 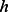 for the whole image, there will be regions oversmoothed or undersmoothed in the restored image, because a single filter parameter 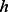 is not optimal for all the patches in the image. As the nonlocal TV norm defined in (27), we will calculate the filter parameter 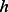 adaptively using local information and get the local 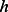 for every pixel in the image.

Inspired by local regularization in [21], we define the local power as:
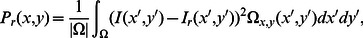
(29)and 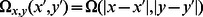 is a normalized smoothing window, here we use a Gaussian window. 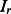 is the expected image, 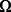 is a region to calculate the local power centered at 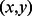.

Then we use the local power to calculate the local 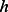 as follows:
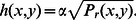
(30)


The advantage of localizing the filter parameter 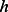 is that it can control the denoising process over image regions according to their content, the smooth regions have average 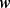 between there neighbors, texture and edge regions have big 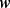 only when the patches are similar. Besides, we do not have to know or estimate the noise condition. In this paper, we use a preprocessed oversmoothed image 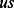 as the expected image instead of the mean of the patch to get more accurate results. The oversmoothed image is obtained by a standard TV model using a large regularization parameter.

By applying the above adaptive nonlocal regularization, the algorithm 1 can be reformed as the following algorithm, where 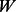 is the function to calculate the weights between points and 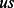 is the preprocessed oversmoothed image:

Algorithm 2: Adaptive nonlocal alternating Bregman iterative method for image restoration.
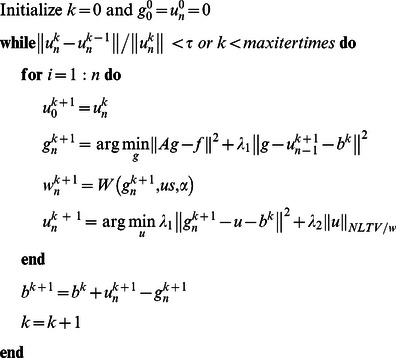



## Experiments

In this section, we present some experimental results of the proposed alternating Bregman method and the adaptive nonlocal alternating Bregman method, and compare them with the operator splitting TV regularization [10], NLTV based BOS algorithm, FTVd algorithm [38], FAST-TV [30] and ForWaRD algorithm [39]. The ForWaRD algorithm is a hybrid Fourier-wavelet regularized deconvolution (ForWaRD) algorithm that performs noise regularization via scalar shrinkage in both the Fourier and wavelet domains.

We use the conjugate gradient method to solve the first subproblem in algorithm 1 and algorithm 2, use the Chambolle's projection algorithm to solve the second subproblem in algorithm 1 and the nonlocal version of the Chambolle's projection algorithm in algorithm 2. In algorithm 1 and algorithm 2, we set 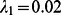 and 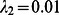 by experiment results, the inner iteration times 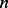 can be set as 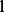 or 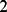 and the stopping condition is 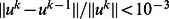. There are lots of work on determining the parameters in the regularization [40], [41], but this work is out of the scope of this paper and we will get in to it later. In algorithm 2, we set the patch size as 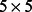, searching window as 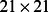 and set the Gaussian variance parameter as 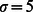 to calculate the local variance. And we set the nonlocal parameter factor as 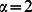. For the operator splitting method, the regularization parameter is set as 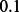. For the NLTV based BOS method, the regularization parameter is set as 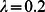, searching window is set as 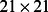, the patch size is set as 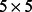 and the nonlocal filter parameter is set as 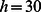. For the FTVd algorithm, we set 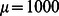. For the FAST-TV, we set 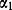 as 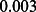 and 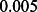 according to the degeneration of the image. And the best valve of 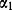. For the ForWaRD algorithm, we set the threshold as 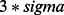, 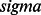 is the standard deviation of the noise, and the regularization parameter is set to 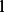.

**Figure 1:**
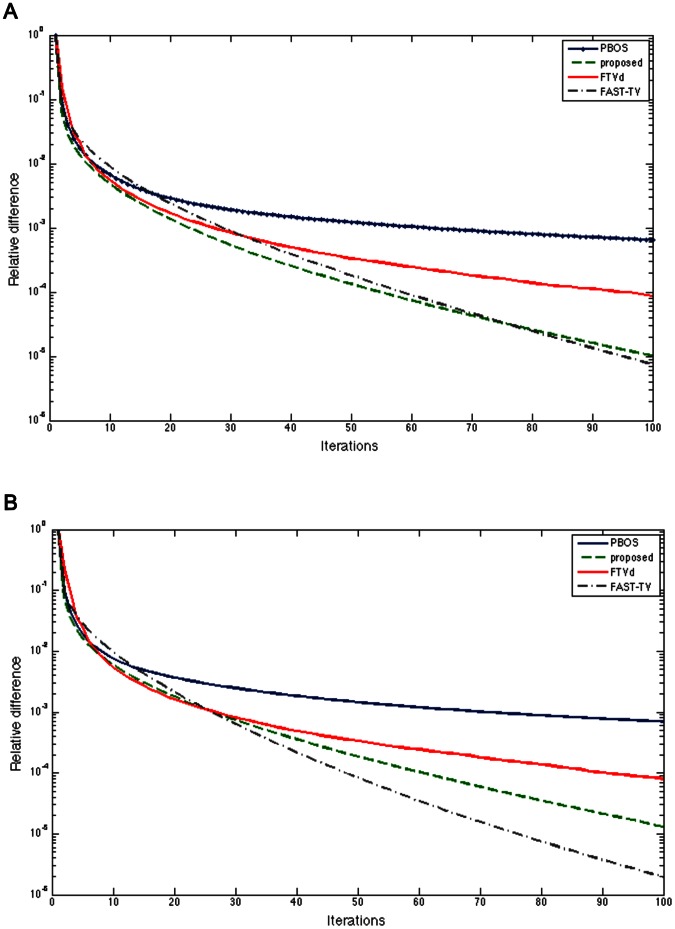
Convergence speed between algorithm 1, operator splitting TV, FTVd and FAST-TV. A. 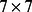 Gaussian kernel with 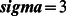 and gaussian noise with 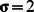 B. 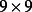 average kernel and gaussian noise with 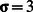. The figure shows the convergence speed between four methods using the Cameraman image and two different blur kennels. Axis X stands for the iteration times, Axis Y stands for the relative difference between restored images in two iterations, that is 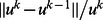.

First, we compare the convergence speed of the proposed algorithm 1 with the preconditioned BOS algorithm, the FTVd algorithm and the FAST-TV algorithm in Figure 1. We can find that the proposed algorithm 1 converge faster than the other three methods at first, and still much faster than the preconditioned BOS algorithm and the FTVd algorithm later, close or a little bit slower than the FAST-TV at the end of the iterations. Usually, the stopping condition of the relative difference is set to 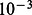 or 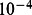. Thus, the proposed algorithm 1 can reach the stopping condition with fewer iterations than other algorithms. In terms of the computation time, the FTVd algorithm is the fastest owning to its strategies and code optimization. And the proposed algorithm 1 is faster than the operator splitting algorithm and the FAST-TV algorithm. As for the nonlocal methods, convergence can not be promised after some iterations, so we compare the computation time between these methods. As the computation of the nonlocal weights, the nonlocal based algorithms cost more computing time than the non nonlocal ones. The NLTV based BOS algorithm stops with 25 steps for 180 seconds, and the preconditioned NLTV based BOS algorithm stops with 8 steps for 75 seconds, however the proposed algorithm 2 stops with 5 steps for only 47 seconds.

**Figure 2:**
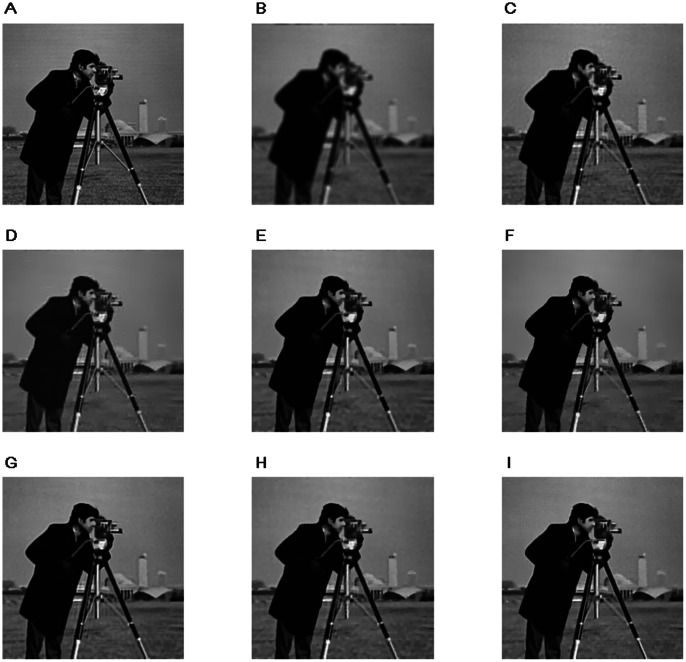
Restoration results on a 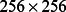 Cameraman image degraded by a 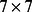 Gaussian kernel with 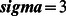 and a gaussian noise with 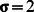
. A. Original Image B. Degraded Image C. Operator Splitting TV D. ForWard E. FTVd F. FAST-TV G. NLTV+BOS H. Algorithm 1 I. Algorithm 2.

**Figure 3:**
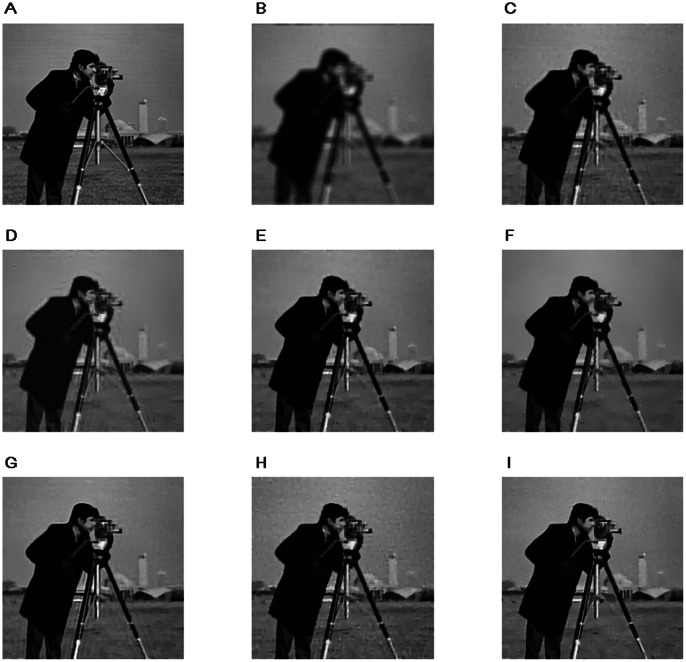
Restoration results on a 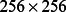 Cameraman image degraded by a 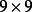 average kernel and a gaussian noise with 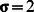
. A. Original Image B. Degraded Image C. Operator Splitting TV D. ForWard E. FTVd F. FAST-TV G. NLTV+BOS H. Algorithm 1 I. Algorithm 2.

Next, we show some image restoration results of these methods to illustrate the effectiveness of the proposed algorithms. We use the classical Cameraman image, so as to be comparable to other image restoration works. The Cameraman image can be found at http://www.imageprocessingplace.com/root_files_V3/image_databases.htm. Figure 2 and Figure 3 show the restoration results on the Cameraman with two kind of blur kernels. We can see from the results that, the ForWard method can get a good restoration result when the image is not slightly blurred, but poor on the heavily blurred situation, besides the ForWard method can not restore edges clearly. The restoration results of the operator splitting TV method have artificial strips which affect the visual appearance of the restored images. FTVd method and FAST-TV can effectively remove noise from the degenerated images, and have higher PSNRs than the ForWard method and the operator splitting TV method, however, a lot of details are also smoothed. The results of the proposed algorithm 1 have good visual appearance, clear edges and preserved image contrast. The NLTV based BOS method (the preconditioned BOS has almost the same result) and the proposed algorithm 2 have better restoration results than the not nonlocal ones, and the proposed algorithm 2 have more details restored and a higher PSNR.

The Table 1 and Table 2 shows the restoration results on 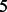 different images and 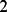 different degradation situations. We can see that, the PSNR and SSIM of the proposed algorithms are generally higher than the methods being compared.

**Table 1:** PSNR and SSIM results of the methods on five different images with a 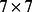 Gaussian kernel with 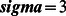 and gaussian noise 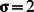.

Image	Blur/noise variance	PSNR	SSIM
	Operator splitting	25.81	0.701
	ForWARD	26.18	0.682
	FTVd	25.71	0.818
Cameraman	FAST-TV	26.20	0.813
	NLTV+BOS	26.00	0.830
	Algorithm 1	26.40	0.830
	Algorithm 2	27.21	0.832
	Operator splitting	23.52	0.667
	ForWARD	23.79	0.701
	FTVd	23.12	0.667
Barbara	FAST-TV	23.28	0.706
	NLTV+BOS	23.59	0.732
	Algorithm 1	23.65	0.701
	Algorithm 2	23.89	0.734
	Operator splitting	25.88	0.716
	ForWARD	25.61	0.654
	FTVd	25.49	0.762
Man	FAST-TV	25.65	0.755
	NLTV+BOS	25.99	0.780
	Algorithm 1	26.06	0.781
	Algorithm 2	26.37	0.786
	Operator splitting	27.60	0.813
	ForWARD	27.30	0.799
	FTVd	26.21	0.825
Boats	FAST-TV	26.78	0.821
	NLTV+BOS	28.01	0.843
	Algorithm 1	27.45	0.841
	Algorithm 2	28.05	0.844
	Operator splitting	27.29	0.799
	ForWARD	27.08	0.791
	FTVd	26.46	0.824
Lenna	FAST-TV	27.09	0.812
	NLTV+BOS	27.52	0.836
	Algorithm 1	27.54	0.832
	Algorithm 2	28.00	0.836
	Operator splitting	26.02	0.739
	ForWARD	25.99	0.725
	FTVd	25.40	0.779
Average	FAST-TV	25.80	0.781
	NLTV+BOS	26.22	0.804
	Algorithm 1	26.22	0.797
	Algorithm 2	26.70	0.806

**Table 2:** PSNR and SSIM results of the methods on five different images with a 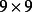 average kernel and gaussian noise 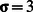.

Image	Blur/noise variance	PSNR	SSIM
	Operator splitting	24.68	0.691
	ForWARD	24.54	0.649
	FTVd	25.18	0.789
Cameraman	FAST-TV	24.87	0.781
	NLTV+BOS	25.16	0.755
	Algorithm 1	25.21	0.760
	Algorithm 2	25.98	0.777
	Operator splitting	23.12	0.639
	ForWARD	23.22	0.660
	FTVd	23.12	0.683
Barbara	FAST-TV	23.18	0.657
	NLTV+BOS	23.21	0.677
	Algorithm 1	23.16	0.680
	Algorithm 2	23.66	0.706
	Operator splitting	24.34	0.618
	ForWARD	23.98	0.588
	FTVd	24.12	0.701
Man	FAST-TV	24.06	0.675
	NLTV+BOS	24.51	0.702
	Algorithm 1	24.59	0.706
	Algorithm 2	24.90	0.712
	Operator splitting	25.82	0.748
	ForWARD	25.40	0.736
	FTVd	24.87	0.780
Boats	FAST-TV	25.11	0.762
	NLTV+BOS	26.15	0.783
	Algorithm 1	25.73	0.778
	Algorithm 2	26.33	0.783
	Operator splitting	26.01	0.766
	ForWARD	25.57	0.746
	FTVd	25.52	0.771
Lenna	FAST-TV	25.67	0.765
	NLTV+BOS	26.22	0.776
	Algorithm 1	26.03	0.766
	Algorithm 2	26.63	0.783
	Operator splitting	24.79	0.692
	ForWARD	24.54	0.676
	FTVd	24.56	0.730
Average	FAST-TV	24.58	0.728
	NLTV+BOS	25.05	0.739
	Algorithm 1	24.94	0.738
	Algorithm 2	25.50	0.752

### Conclusions

In this paper, we propose a Bregman iteration based total variation image restoration algorithm. We split the restoration problem into a three step iteration process, and these steps are all easy to solve. In addition, we propose a nonlocal regularization under the framework of the proposed algorithm using a point-wise local filter parameter, and a method to adaptively determine the filter parameter. Experiments show that the algorithm converges fast and the adaptive nonlocal regularization method can obtain better restoration results. In the future, we will consider the weights updating problem in a theoretical way and apply the proposed algorithms for other regularization problems such as compressed sensing.
